# Efficacy and safety of loxoprofen hydrogel patch versus loxoprofen tablet in patients with ankylosing spondylitis: A 4-week randomized, open-label study

**DOI:** 10.3892/br.2019.1232

**Published:** 2019-07-23

**Authors:** Meida Fan, Shuangyan Cao, Liudan Tu, Qiujing Wei, Riwei Yuan, Xuefeng Li, Jieruo Gu

Biomed Rep 10:331-336, 2019; DOI: 10.3892/br.2019.1209

After the publication of the above paper, the authors noted that the affiliation for the first named author (Meida Fan) was presented incorrectly: Specifically, both of the first two affiliations should have been assigned to Meida Fan, and furthermore, these affiliations should have been presented the other way around. Therefore, the authors' names and affiliations in this paper should have been presented as follows:

MEIDA FAN^1,2^, SHUANGYAN CAO^1^, LIUDAN TU^1^, QIUJING WEI^1^, RIWEI YUAN^1^, XUEFENG LI^3-5^ and JIERUO GU^1^

^1^Department of Rheumatology and Immunology, The Third Affiliated Hospital of Sun Yat-Sen University, Guangzhou, Guangdong 510630; ^2^Department of Rheumatology and Immunology, Nanfang Hospital, Southern Medical University, Guangzhou, Guangdong 510515; ^3^The Sixth Affiliated Hospital of Guangzhou Medical University, Qingyuan People's Hospital, Sino-French Hoffmann Institute, School of Basic Medical Sciences, Guangzhou Medical University, Guangzhou, Guangdong 511436; ^4^Shenzhen Luohu People's Hospital, The Third Affiliated Hospital of Shenzhen University, Shenzhen, Guangdong 518001; ^5^Key Laboratory of Regenerative Biology, Guangdong Provincial Key Laboratory of Stem Cell and Regenerative Medicine, South China Institute for Stem Cell Biology and Regenerative Medicine, Guangzhou Institutes of Biomedicine and Health, Chinese Academy of Sciences, Guangzhou, Guangdong 510530, P.R. China

The authors regret that this error with the author affiliations for Meida Fan was not noticed prior to the publication of their paper, and apologize for any inconvenience caused.

